# Supplementary material for: A complete tool set for molecular QTL discovery and analysis
Source: Nat Commun. 2017 May 18;8:15452. doi: 10.1038/ncomms15452 (PMC5454369; doi:10.1038/ncomms15452)
Supplement: Supplementary Information — Supplementary Figures, Supplementary Table, Supplementary Notes and Supplementary References [file ncomms15452-s1.pdf]

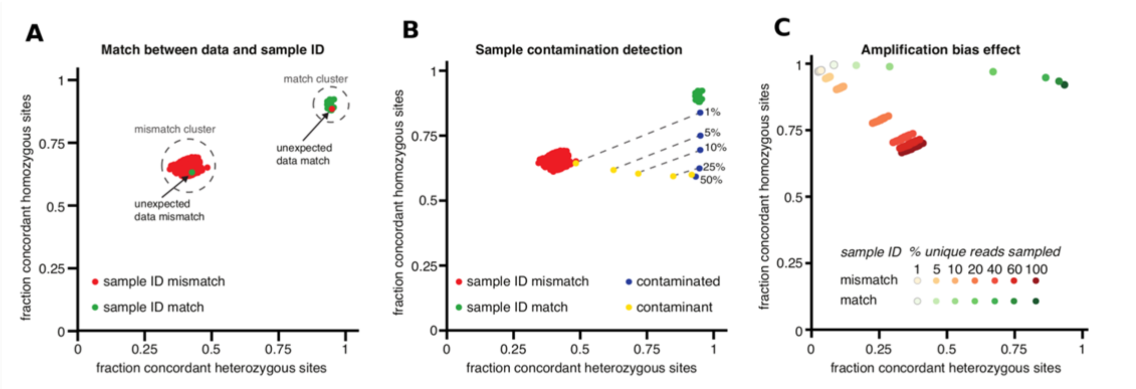

Supplementary Figure 1: Possible problems spotted by sequence and genotype data matching. Following the same graphical representation than in Supplementary Fig. 3, this figure shows three potential problems that the mbv mode of QTLtools can detect. Panel A shows two mislabeling scenarios: (i) a pair of samples with matching IDs shows very poor concordance suggesting that the sequence and genotype data do not originate from the same individual and (ii) a pair of samples with mismatching IDS shows very good concordance meaning that the sequence and genotype data come from the same individual. Panel B shows the effect of 1% to 50% contamination on the concordance measures: as you increase the amount of contamination, the concordance at homozygous genotypes decreases. Panel C shows the effect of PCR amplification bias simulated here by resampling different fractions (1% to 100%) of unique reads in the original BAM file: this decreases significantly concordance at heterozygous genotypes.

1

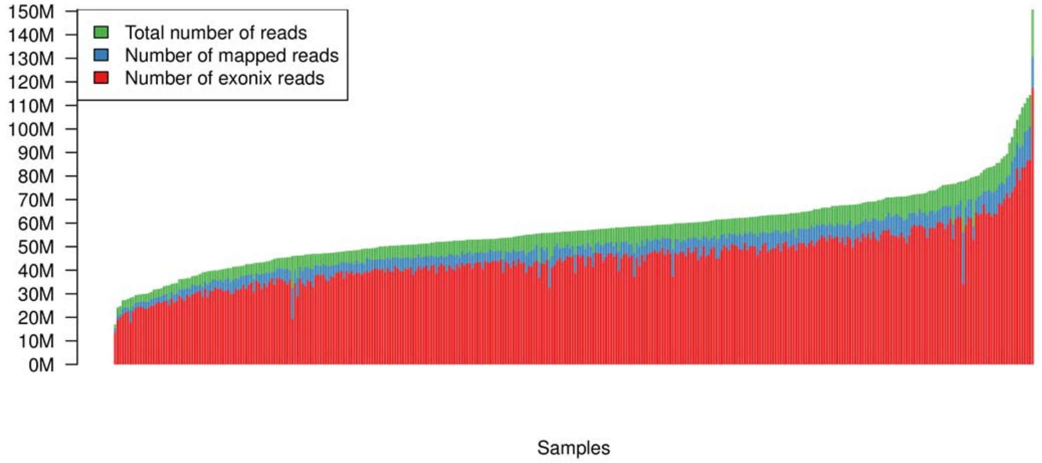

Supplementary Figure 2: Quality Control of the sequence data. For each of the 258 Geuvadis samples (on the x-axis), this plot shows the total numbers of reads in green, the number of reads passing all QC filters described in Supplementary Note 1 in blue and the number of exonic reads in red (all on the y-axis).

2

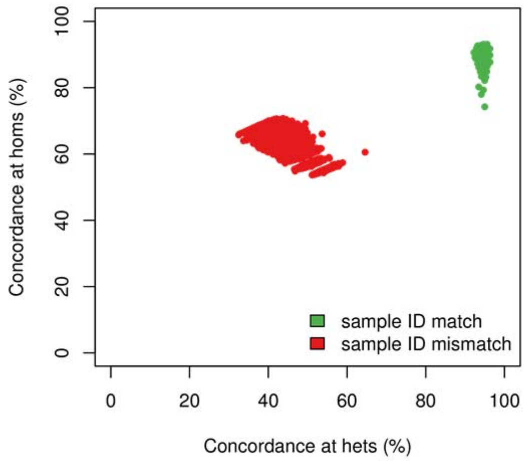

Supplementary Figure3: Matching sequence and genotype data. For all possible pairwise combinations between sequenced and genotyped samples in Geuvadis (n = 358 x 358 = 128,164 pairs), the concordance between the sequence and genotype data is measured separately for homozygous (y-axis) and heterozygous (x-axis) genotypes (Supplementary Note 2). All pairs with matching sample IDs are shown in green while all pairs with different sample IDs are shown in red. When the sample IDs match, the concordance measures are high meaning that there is no mislabeling between the sequence and genotype data.

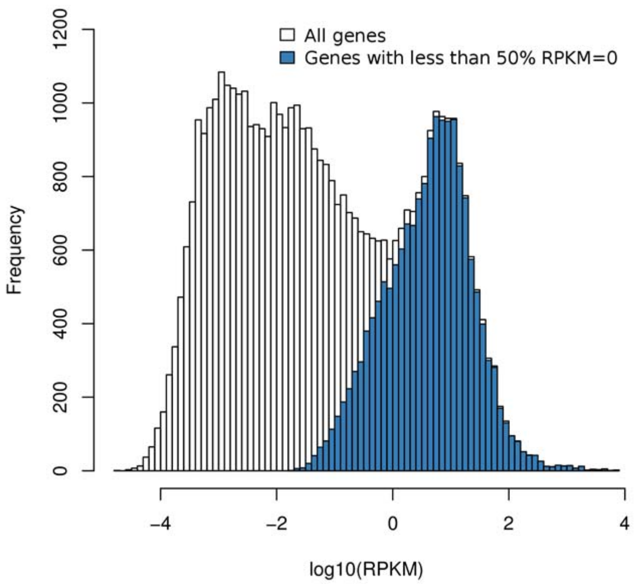

Supplementary Figure 4: Gene expression quantification. We measured gene expression levels as RPKMs (Reads Per Kilobase per Million mapped reads; Supplementary Note 3) for all genes reported in GENCODE v19 [**1**] (shown with white bars). Then, we only kept the subset of genes with non-zero quantifications in at least 50% of the Geuvadis samples (shown with dark blue bars), resulting in a set of 22,147 genes kept for downstream analysis.

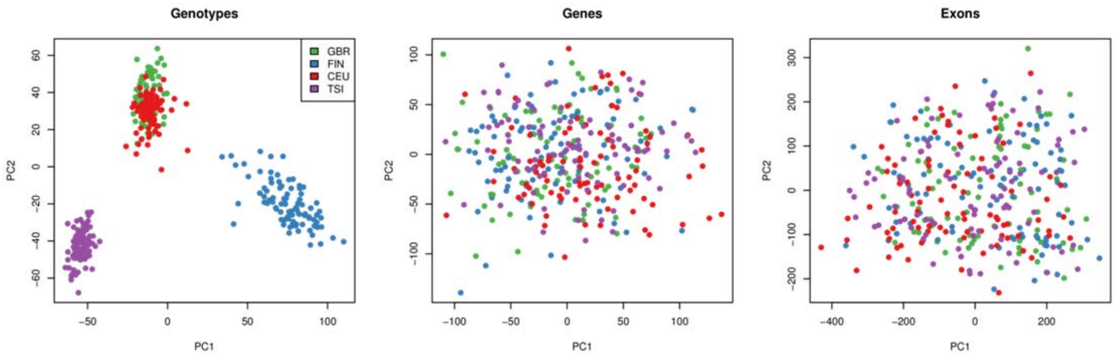

Supplementary Figure 5: Stratification of the genotype and sequence data. Scatter plots of sample coordinates on the first (x-axis) and second (y-axis) principal components (PC) for genotype data, gene quantifications and exon quantifications (from left to right). Colors here are just indicative of the sample various ancestries.

4

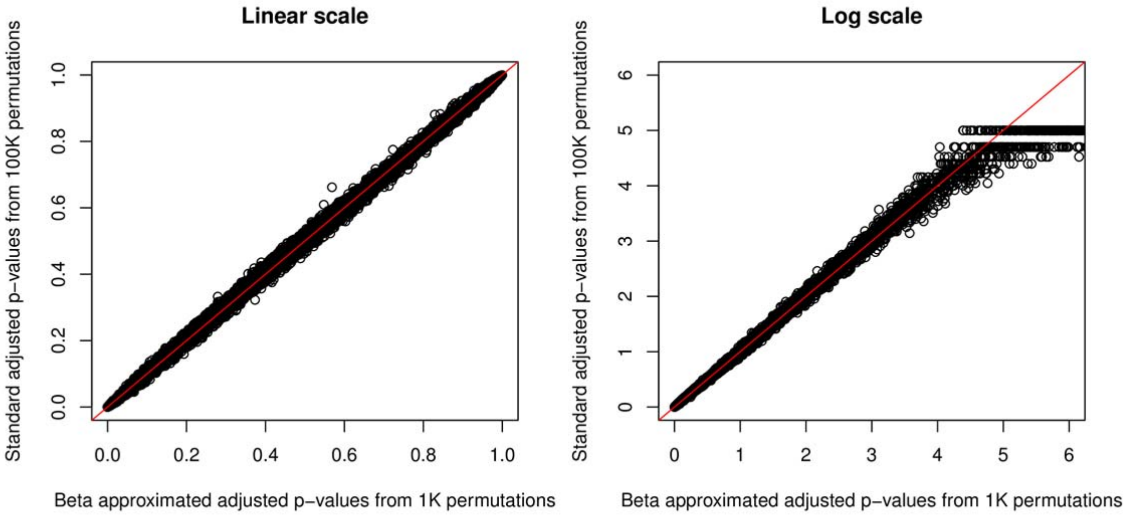

Supplementary Figure 6: Beta approximation of the permutation process. These two scatter plots compare the P-values adjusted for multiple genetic variants being tested in *cis* via (i) the beta approximation from 1,000 permutations (x-axis) and (ii) the direct method from 100,000 permutations (y-axis). The comparison is made on linear (left panel) and log scales (right panel); in both cases for the Geuvadis data set (see Methods). The red diagonal shows idealistic correspondence between both sets of adjusted P-values.

5

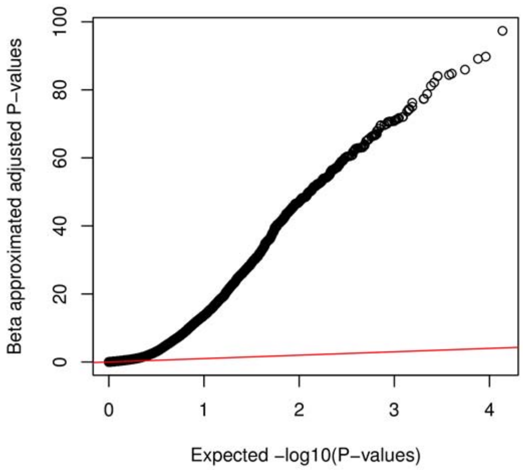

Supplementary Figure 7: Adjusted P-value range. This Quantile-Quantile plot compares the expected (x-axis) and observed (y-axis) distributions of adjusted P-values via beta approximation on the Geuvadis data set. The smallest observed P-value reaches 4.62 x 10^{-98}.

6

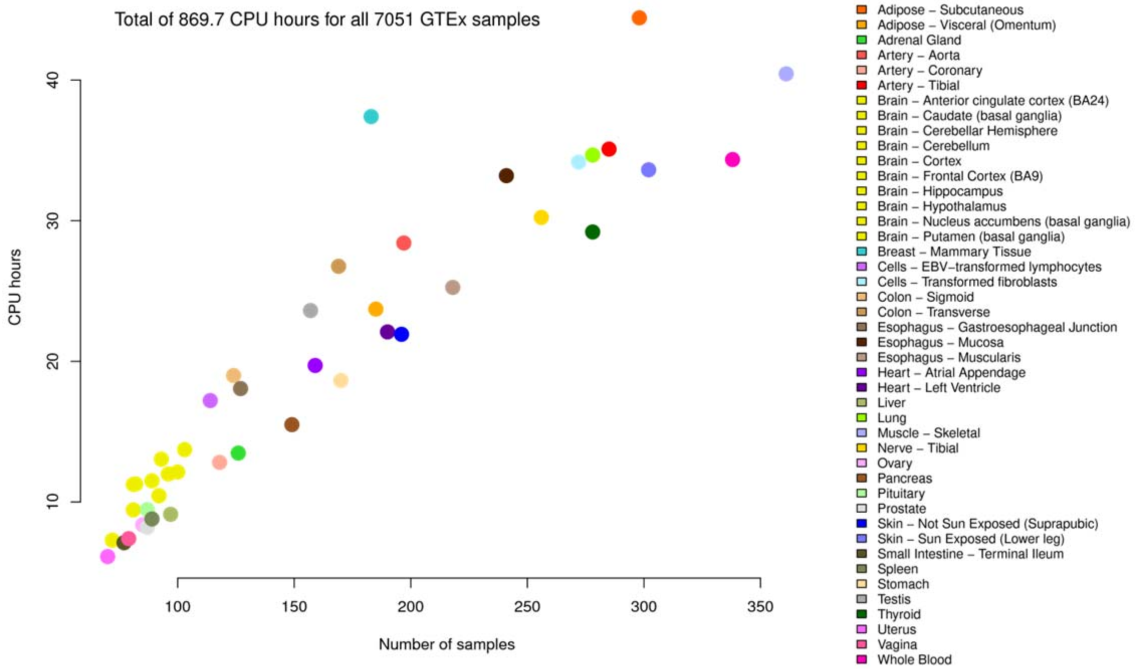

Supplementary Figure 8: Running times for eQTL mapping in cis for the entire GTEx v6p data set. This plot shows the running times required to map eQTL in cis for each of the 44 tissues of the GTEx v6p study [2] as a function of the sample sizes.

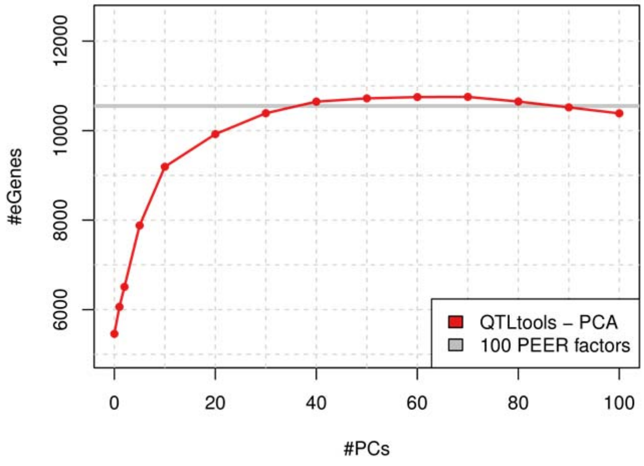

Supplementary Figure 9: Effect of the number of PCA-derived covariates on the discoveries. This plot shows in red the number of genes with at least an eQTL (i.e. eGenes) discovered in Geuvadis (y-axis) as a function of the number of Principal Components (PCs; 0 to 100) derived from gene expression data in order to correct for technical variance (y-axis). Beside this, the grey line shows the outcome when using 100 PEER factors [3] instead of PCs; a widely adopted method in the field.

7

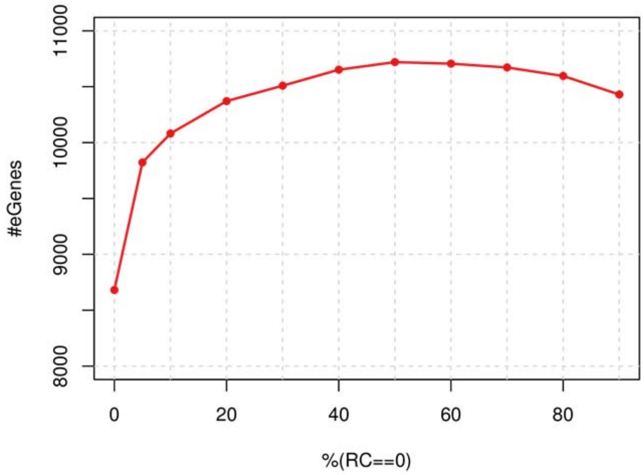

Supplementary Figure 10: Effect of phenotype filtering on the discoveries. This plot shows in red the number of eGenes discovered in Geuvadis (y-axis) as a function of the filtering criterion used to exclude poorly quantified genes (x-axis). Specifically, we measured the percentage of individuals per gene not being quantified; that is with a read count equal to 0 and filtered genes accordingly to this percentage from 0% to 90%.

8

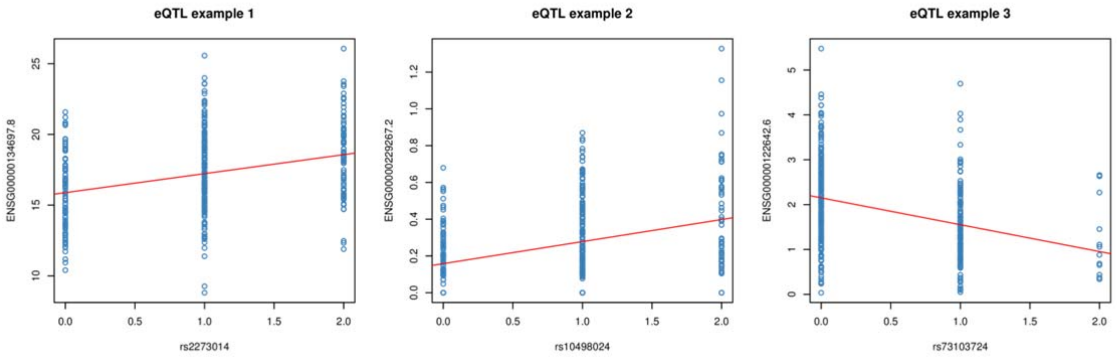

Supplementary Figure 11: Three specific eQTL examples. Three different examples of significant eQTLs discovered in Geuvadis. The raw genotype and sequence data were extracted using the QTLtools extract mode and plotted with the R/plot function. Each plot shows the effect of genotype dosages at a given eQTL on gene expression measured via RPKM. Regression lines are shown in red.

9

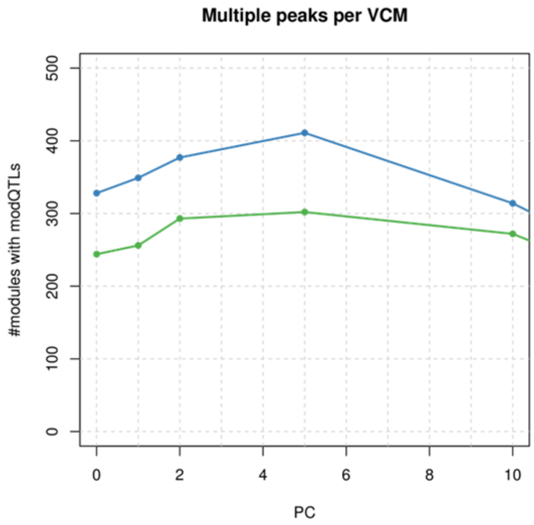

Supplementary Figure 12: Respective performance of two phenotype grouping methods. This plot shows the numbers of eGenes discovered in the histone modification data set (Supplementary Data 2) as a function of the number of Principal Components (PCs) used to correct for technical variance. It is shown the performance of two different ways of aggregating the signal of multiple histone marks belonging in the same Variable Chromatin Module (VCM [4]) at the QTL mapping level (see Methods) by using either the extended permutation scheme (in blue) or Principal Component Analysis (in green).

10

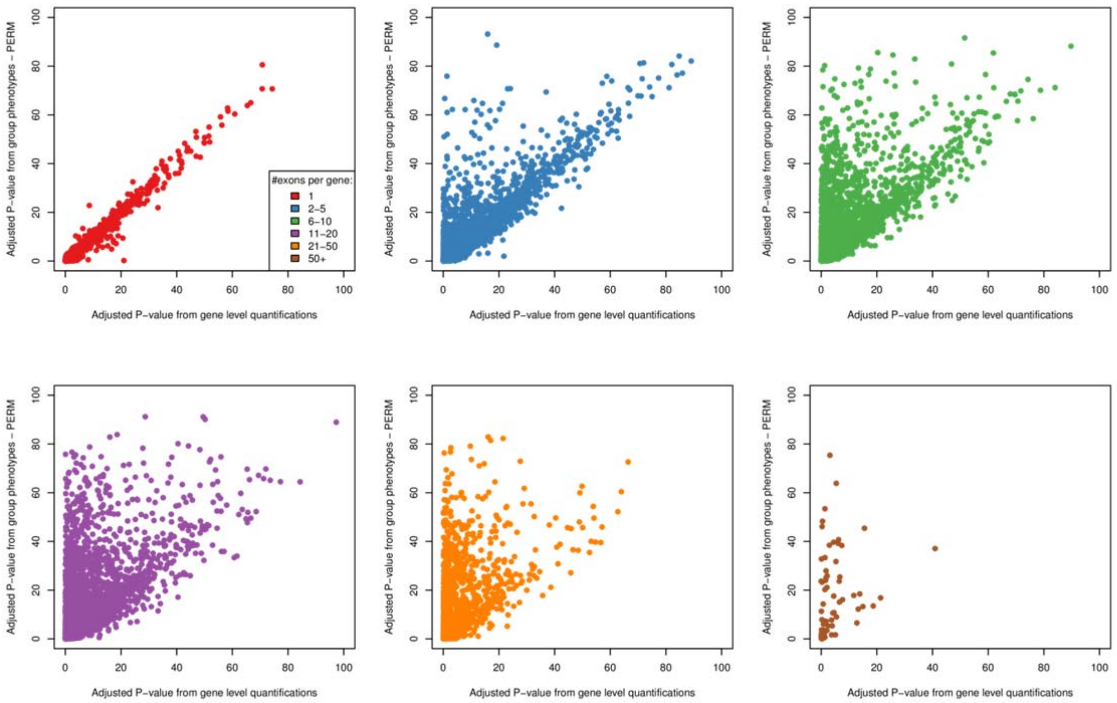

Supplementary Figure 13: Comparison between gene quantification and phenotype grouping. These six scatter plots compare on a per gene basis the –log10 of the nominal P-values obtained when running the QTL mapping on gene level quantifications (x-axis) or by using phenotype grouping (extended permutation scheme; y-axis; see main Methods section). Adjusted P-values have been compared in six categories, depending on the number of exons the genes contain: 1 (in red), 2 to 5 (in blue), 6 to 10 (in green), 11 to 20 (in purple), 21 to 50 (in orange) or more than 50 (in brown).

11

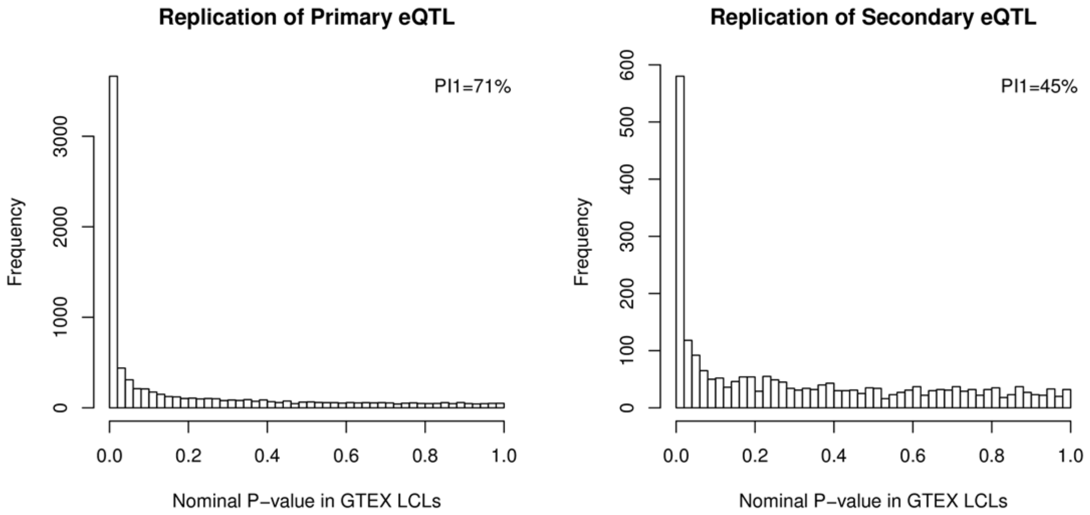

Supplementary Figure 14: Replication of eQTLs. These two histograms show the nominal p-value distributions in GTEx [2] for both primary (left panel) and secondary (right panel) eQTLs discovered in Geuvadis. For each, we estimated the percentages of eQTLs that are significant in GTEx via R/qvalue (i.e. PI1 statistic; Supplementary Note 6).

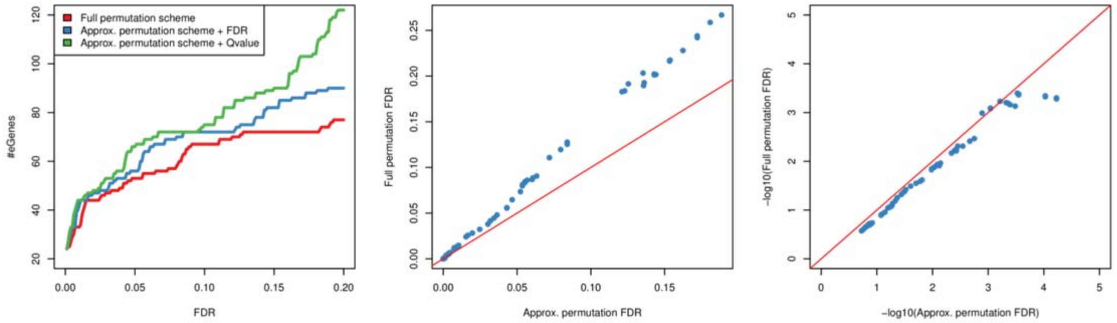

Supplementary Figure 15: Performance of the approximation for trans QTL mapping. The left panel shows the number of genes with at least a significant eQTL in *trans* for 3 different configurations: (i) the full permutation scheme (in red; see main Methods section), (ii) the approximation scheme using either the BH (in blue; Benjamini and Hochberg [5]) or ST (in green; Storey et Tibshirani [6]) FDR procedures to correct for the number of genes being tested. The two other panels compare the FDR estimates on a per gene basis obtained by (i) and (iii) on linear (middle panel) and log (right panel) scales.

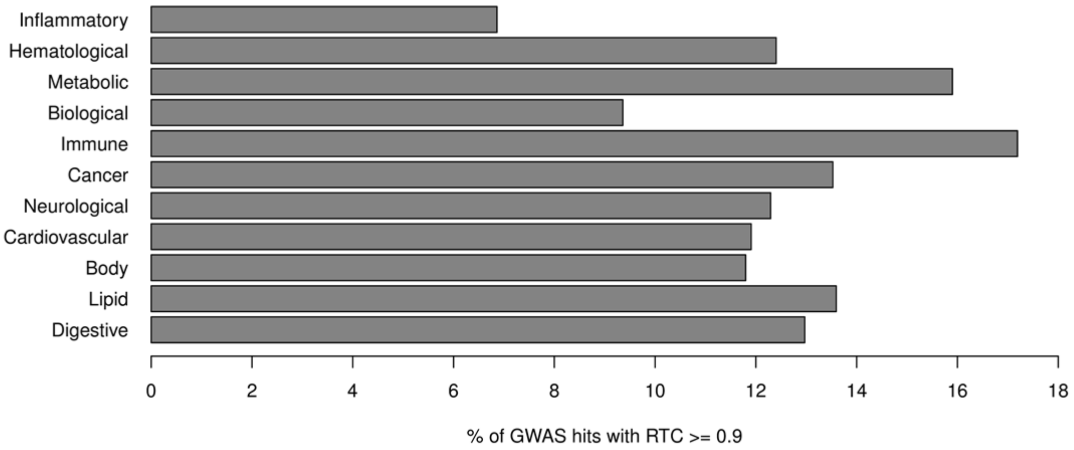

Supplementary Figure 16: RTC results. Percentages of hits of the NHGRI catalog being tagged by gene level eQTLs discovered in Geuvadis (i.e with a RTC score >= 0.9; Supplementary Note 7). The GWAS hits have been categorized into multiple disease ontologies (FTO).

Mode	Description	Running time in hours
bamstat	QC the sequence data	461
match	Check that sequence data matches genotype data	40
quant	Quantify exon and gene expression	115
pca	Perform PCA on phenotype and genotype data	1
cis (genes)	Map gene level eQTLs from gene quantifications in cis	32
cis (groups)	Map gene level eQTLs from exon quantifications in cis	210
cis (cond.)	Map multiple eQTLs per gene in cis	4
trans (full)	Map eQTLs in trans	450
trans (approx.)	Map quickly eQTLs in trans	7
rtc	Integrate eQTLs with GWAS hits	2
func	Integrate eQTLs with functional annotations	5
TOTAL		1327

Supplementary Table1: Summary table of the QTLtools tasks performed on Geuvadis. Each row gives the name of the mode used, a short description of it and the running time needed to be run on the whole data.

Supplementary Note 1: Controlling the quality of the sequence data

To ensure good quality of the sequence data, QTLtools parses a single BAM file and counts the total number of alignments passing all these filtering criteria:

1. The alignment is not tagged as unmapped (BAM_FUNMAP).
2. The alignment is not tagged as secondary alignment (BAM_FSECONDARY).
3. The alignment is not tagged as failing QC (BAM_FQCFAIL).
4. The alignment has a mapping quality (MAPQ) above a given threshold.

In the case of pair-end reads, they also need to pass this additional set of QC criteria:

5. Both reads in a pair need to pass the QC filters 1-4.
6. Both reads in a pair need to be on opposite strand.

In addition to this, QTLtools also counts the number of reads falling within some known annotation such as GENCODE [**1**] for RNA-seq and ENCODE [**7**] for ChIP-seq. Note that for ChIP-seq, it is recommended to use an annotation file that has been generated for the same molecular assay and cell type. In practice, QTLtools requires the annotations to be specified with either a GTF or a BED file. Then, percentages of mapped and annotated reads can be computed in order to detect major problems in the sequence data; an outlier being an evidence of a problem occurring at library preparation or sequencing. Note here that it is difficult for us to provide precise guidelines in term thresholds to be used in order to decide on the inclusion or exclusion of a sample since it highly depends on the sequencing protocols used to generate the data and therefore requires decision to be made on a case-by-case basis. For instance, in the Geuvadis data, we find two samples for which the mapped and annotated reads is relatively low compared to the other samples, but not enough for us to discard them from downstream analysis (Supplementary Fig. 2).

<u>Supplementary Note 2:</u> Checking that sequence data matches genotype data

To make sure that both the sequence and genotype data match, QTLtools takes as input a VCF file containing the genotype data for one or multiple samples and a BAM file with the mapped sequences of a molecular assay (e.g. RNA-seq or ChIP-seq). It first piles up sequencing reads at each single-nucleotide-variants (SNVs) site in the VCF file. It then discards poorly covered SNVs (as defined by a minimal-coverage parameter) and measures, for each individual in the VCF, the proportions of heterozygous and homozygous genotypes for which both alleles are captured by the sequencing reads (BAM file). We obviously expect here a very high correspondence between sequence and genotype data. To do so, QTLtools first enumerates all polymorphic sites passing various quality filters (such as minimal coverage, imputation quality, minor allele frequency, etc …). Then, for each individual in the VCF file, it counts:

- **A.** The number of homozygous genotypes REF/REF covered by reads carrying only the REF allele.
- **B.** The number of homozygous genotypes REF/REF covered by at least one read carrying only the ALT allele.
- **C.** The number of homozygous genotypes ALT/ALT covered by reads carrying only the ALT allele.
- **D.** The number of homozygous genotypes ALT/ALT covered by at least one read carrying only the REF allele.
- **E.** The number of heterozygous genotypes REF/ALT covered by reads carrying either the REF or ALT alleles.
- **F.** The number of heterozygous genotypes REF/ALT covered by reads carrying only the REF allele <u>or</u> the ALT allele.

Here REF and ALT denote the reference and alternative alleles carried by an individual at a given position: REF means that a allele match the reference genome, while ALT means that it differs. Then, QTLtools computes the two following concordance measures:

1. At homozygous genotypes by $\mathbf{C_0 = (A+B)/(A+B+C+D)}$
2. At heterozygous genotypes by $\mathbf{C_1 = E/(E+F)}$

Finally, these measures are reported for each individual in the output file together with other secondary statistics that are not worth to mention here. A good practice to rapidly identify the set of individuals for which there is a match between the sequence and the genotype data is to visualize the two concordance measures on a scatter plot (similarly to Hoen et al. [6]). We applied this method on all available Geuvadis RNA-seq BAM files and observed that matches appear as points close to 100% concordance for both measures whereas mismatches as points far from this optimal position (Supplementary Fig. 3). Points with unexpected locations can suggest various issues in the sequence data. To illustrate this, we simulated 3 different possible and realistic sequencing issues on a subset of 20 Geuvadis BAM files. First, we simulated sample mislabeling by swapping randomly two sample IDs and observed the effect on the scatter plot (Supplementary Fig. 1A). Second, we simulated sample contamination by adding to a particular BAM file (i.e. the contaminated sample) various percentages (1% to 50%) of another BAM file (i.e. the contaminant sample) to illustrate the ability of our method to detect such cases (Supplementary Fig. 1B). Finally, we simulated

amplification biases by extracting all unique reads (i.e. non-duplicated reads) in a BAM file and then by sampling from various subsets of them (1% to 100% of the unique reads) enough reads to match the original BAM file size. This allowed us to characterize the performance of our method to detect amplification biases (Supplementary Fig. 1C).

Supplementary Note 3: Quantifying gene expression

For convenience, QTLtools can also quantify gene expression given a RNA-seq BAM file and a gene annotation GTF file such as those provided by GENCODE [1]. For the moment, the quantification module is only designed to handle RNA-seq data and cannot process optimally ChIP-seq data since this task requires additional processing related to fragment length estimation. Specifically, QTLtools quantifies in turn each exon listed in the GTF file by counting the number of overlapping reads that pass the multiple QC criteria described above (Supplementary Note 1). Then, it offers functionalities to normalize the resulting read counts by the library size and to sum up the read counts per gene. As output, both raw read counts or counts per kilobase per million of reads (RPKM) are reported in separate BED files. QTLtools can either quantify one sample at a time or multiple of them simultaneously in order to directly build a quantification matrix. In addition, it includes options to filter reads exhibiting too many mismatches with the reference genome and merge overlapping exons when necessary to avoid double counting some sequencing reads.

Supplementary Note 4: Performing Principal Component Analysis

An important step in any molecular QTL study relies on studying the internal structure of the data that best explains its variance. In other words, it is crucial to study population stratification prior to any other analysis. Principal Component Analysis (PCA) is a well-established method to achieve this task and capture multiple data features that need to be accounted for in any downstream analysis. For instance, a PCA on genotype data is often used to detect and quantify population structure (i.e. population-specific variations in allele frequencies) [7], while a PCA on gene expression is known to capture technical variance (e.g. date of sequencing, library preparation, etc …). In practice, the first principal components (PCs) of each can be used to either exclude outliers (i.e. data point too distant from sample distribution) or capture various confounding factors that can boost discovery power when accounted for in association testing. QTLtools allows performing PCA on both genotype and phenotype data. When relevant, the input data can be centered, scaled and trimmed for MAF and LD in the case of genotype data. When applied on a trimmed version of the Geuvadis genotype data, the PCA performed with QTLtools gives strictly equivalent results than what can be obtained with the R/princomp or R/prcomp functions but it is ~20 times faster (data not shown) to get the results. From this analysis, we could not see any clear evidence of unexpected stratification in both gene expression and genotype data (Supplementary Fig. 5).

Supplementary Note 5: Covariates in association testing

To correct for covariates (user- or PCA-derived) in association testing, QTLtools residualizes all the phenotype data (e.g. expression levels) for covariates using linear regressions as implemented in the R/lm function. Prior to any association testing, it basically produces a new phenotype matrix with quantifications that are independent of any of the covariates. In practice, we always run a PCA on the phenotype data before the QTL mapping stage and produce from this multiple sets of covariates including different numbers of PCs. We then repeat the QTL mapping stage across these multiple

sets of covariates in order to determine the number of PCs that maximizes the number of discoveries. Concerning the genotype data, we systematically use the 3 first PCs to correct for population stratification. In addition to this, QTLtools can also enforce the per-phenotype quantifications to match a normal distribution with mean 0 and standard deviation 1 in order to remove any outlier effects. This is done similarly to the R/rntransform function in the GENABEL package [8].

Supplementary Note 6: Replication of the QTLs in GTEx

To demonstrate that the additional discoveries we made using conditional analysis are genuine, we used gene expression levels and genotype data derived from 115 GTEx Lymphoblastoid Cell Lines (LCL) [9]. To do so, we test for association all gene-variant pairs in *cis* (1Mb window) using the same set of covariates used in the GTEx project and then extracted all those that we identified as significant through conditional analysis in Geuvadis. In total, we could extract ~75% and ~72% of the Geuvadis primary and secondary eQTLs, respectively; the remaining ones involving genetic variants that have not been genotyped in GTEx. We then looked at the P-values in GTEx for all these overlapping eQTLs, produced histograms (Supplementary Fig. 14) and estimated the proportion of them being significant using the PI1 statistic of the R/qvalue package [6]. Note here that the replication rates are constrained by the fact that the sample size in GTEx (n=115) that is much smaller than the one used for discovery (Geuvadis; n=358).

Supplementary Note 7: Integrating QTLs with GWAS

We have previously described a methodology called Regulatory Trait Concordance (RTC) score to assess whether a GWAS variant is tagging the same functional variant as a regulatory variant [10]. In general, a high level of linkage disequilibrium (LD) between a molQTL and a GWAS hit is not enough to claim that they tag the same underlying functional variant. However, if they actually do, we expect that removing the GWAS effect in the molecular phenotype will substantially decrease the molQTL association. The RTC score quantifies exactly this: the change in statistical significance that removing the effect of the GWAS hit has on the molQTL association. And to see how important this change is, we compare it to what we get when we remove the effect of any of the variants within the same genomic region. Specifically, for a molQTL and GWAS variant located in the same genomic region in between two recombination hotspots, we proceed as follows:

1. We create a new molecular phenotype by removing the effect of the GWAS hit. In particular, we residualize the phenotype for the GWAS hit; that is we use as new phenotype the residuals we get when we regress the GWAS hit from the phenotype.
2. We test for association between the molQTL and the new phenotype obtained in step (1).
3. We delimit the genomic region of interest by picking up the interval in between two recombination hotspots that includes both the molQTL and the GWAS hit. Let assume this region contains in total ***L*** variant sites.
4. We create ***L*** pseudo-phenotypes by residualizing each of the ***L*** variants within the same genomic region.
5. We test for association between the molQTL and the ***L*** pseudo-phenotypes which results in a vector of ***L*** P-values that we sort.

6. We determine the rank of the association we get in step (2) within the sorted vector we got in step (5) and divide it by ***L***: this defines a RTC score that ranges from 0 to 1.

When the RTC score is close to 1, it means that accounting for the GWAS hit has a dramatic effect on the molQTL association that is actually larger that doing so for any other variant nearby. This therefore means that it is very likely that the GWAS hit and the molQTL actually tag the same underlying functional variant. In contrast, when the RTC score is close to 0, this means that accounting for the GWAS hit does not have any major effect on the molQTL association and that they probably tag two distinct functional variants. In practice, we consider that two variants tag the same function when their RTC score is above 0.9.

We run this RTC method on (1) the gene-level eQTL data we got for Geuvadis and (2) the GWAS hits from the NHGRI GWAS catalog released on the 2016-06-12 and populated with EFO ontologies for diseases. For each ontology, we measured the number of GWAS hits tagged by at least an eQTL with a RTC score > 0.9, and managed to tag a substantial number of the GWAS hits with eQTLs we discovered in Geuvadis (Supplementary Fig. 17).

Supplementary References

[1] Harrow et al. GENCODE: the reference human genome annotation for The ENCODE Project. Genome Res. 2012 Sep;22(9):1760-74.

[2] Aguet, F. et al. Local genetic effects on gene expression across 44 human tissues. bioRxiv 074450; http://biorxiv.org/content/early/2016/09/09/074450.

[3] Stegle et al. Using probabilistic estimation of expression residuals (PEER) to obtain increased power and interpretability of gene expression analyses. Nat Protoc. 2012 Feb 16; 7(3): 500–507.

[4] Waszak et al. Population Variation and Genetic Control of Modular Chromatin Architecture in Humans. Cell. 2015 Aug 27;162(5):1039-50.

[5] Benjamini & Hochberg. Controlling the False Discovery Rate: A Practical and Powerful Approach to Multiple Testing. Journal of the Royal Statistical Society 1996; 57:289-300.

[6] Storey & Tibshirani. Statistical significance for genomewide studies. Proc Natl Acad Sci. 2003, 100:9440-5.

[5] ENCODE Project Consortium. An integrated encyclopedia of DNA elements in the human genome. Nature. 2012 Sep 6;489(7414):57-74.

[6] Hoen et al. Reproducibility of high-throughput mRNA and small RNA sequencing across laboratories. Nat Biotechnol. 2013 Nov;31(11):1015-22.

[7] Novembre et al. Genes mirror geography within Europe. Nature. 2008 Nov 6; 456(7218): 98–101.

[8] Aulchenko et al. GenABEL: an R library for genome-wide association analysis. Bioinformatics 2007. 23:1294-6.

[9] GTEx Consortium. The Genotype-Tissue Expression (GTEx) pilot analysis: multitissue gene regulation in humans. Science. 2015 May 8;348(6235):648-60.

[10] Nica et al. Candidate causal regulatory effects by integration of expression QTLs with complex trait genetic associations. PLoS Genet. 2010 Apr 1;6(4):e1000895.
